# Relationship Between Myopia and Other Risk Factors With Anxiety and Depression Among Chinese University Freshmen During the COVID-19 Pandemic

**DOI:** 10.3389/fpubh.2021.774237

**Published:** 2021-12-01

**Authors:** Hongmei Zhang, Huijuan Gao, Yun Zhu, Ying Zhu, Weiyu Dang, Ruihua Wei, Hua Yan

**Affiliations:** ^1^Tianjin Key Laboratory of Retinal Functions and Diseases, Tianjin Branch of National Clinical Research Center for Ocular Disease, Eye Institute and School of Optometry, Tianjin Medical University Eye Hospital, Tianjin, China; ^2^Department of Epidemiology and Biostatistics, Tianjin Medical University, Tianjin, China; ^3^Department of Ophthalmology, Tianjin Medical University General Hospital, Tianjin, China

**Keywords:** depression, anxiety, psychological health, myopia, epidemiology

## Abstract

**Purpose:** To investigate the association of myopia and other risk factors with anxiety and depression among Chinese university freshmen during the coronavirus disease 2019 (COVID-19) pandemic.

**Methods:** This cross-sectional study was conducted at the Tianjin Medical University from October 2020 to December 2020. Ophthalmic examination of the eyes was performed by an experienced ophthalmologist. Detailed information on depression, anxiety, and other risk factors was collected *via* the Self-rating Anxiety Scale and Self-rating Depression Scale.

**Results:** The overall prevalence of anxiety and depression in our study was 10.34 and 25.13%, respectively. The prevalence of myopia and high myopia as 92.02 and 26.7%, respectively. There were significant associations between anxiety and spectacle power [odds ratios (OR) = 0.89; 95% CI: 0.81–0.98, *P* = 0.019], sphere equivalent (OR = 0.89; 95% CI: 0.81– 0.98, *P* = 0.025), sleep time (OR = 0.53; 95% CI: 0.35–0.79, *P* = 0.002), and body mass index (OR = 0.93; 95% CI: 0.86–0.99, *P* = 0.047). In the multivariable linear regression models, spectacle power (β = −0.43; 95% CI: −0.68 to −0.19, *P* = 0.001) and sphere equivalent (β = −0.36; 95% CI: −0.60 to −0.11, *P* = 0.005) were negatively associated with anxiety scores, whereas axial length (β = 0.54; 95% CI: 0.02–1.07, *P* = 0.044) was positively correlated with anxiety scores. Every 1 h decrease in sleep time was associated with a 0.12-point increase in depression score.

**Conclusion:** Myopia was associated with anxiety and anxiety scores. The greater the degree of myopia, the higher the anxiety score. However, myopia was not found to be associated with depression. The results highlight the importance of providing psychological support to students with myopia during the COVID-19 pandemic.

## Introduction

Myopia has become a major health problem worldwide owing to its increasing prevalence in the past few decades (1). It is predicted that by 2050, 49.8% of the world population will have myopia and 9.8% will be highly myopic (2). China is one of the countries with a high prevalence of myopia (3). Based on data from a myopia study in Fenghua City, the prevalence of high myopia in China has nearly doubled from 7.9 to 16.6% from 2001 to 2015 (4). In Taiwan, the prevalence of myopia and high myopia in a sample of ~4,000 university freshmen was 91.3 and 23.5% in 1988 and 95.9 and 38.9% in 2005 (5).

The coronavirus disease 2019 (COVID-19) outbreak occurred at the beginning of 2020. The Chinese government took many measures to curb COVID-19, such as the closure of schools with the education of students using online platforms. The increased digital screen time and the overall time spent on near work, together with the decreasing outdoor time, increased the risk of myopia progression in students (6). Accelerated myopic progression has been reported during the COVID-19 pandemic (7–9).

Myopia is not just a refractive error but a leading blinding disorder because of myopic retinopathy and myopia-associated glaucoma, especially in high myopia (2). Vision is an extremely valued sense that affects daily life activities; hence, myopia may face practical difficulties and limitations imposed on sports and career opportunities (10, 11).

University freshmen are special populations that endure a period of great challenges in college entrance examinations, entering new environments, facing risks, and social developmental transition. Previous studies have reported high rates of mental disorders among medical students compared to their peers of the same age (12–14). Depression and anxiety are among the most common mental disorders. Screening for depression or anxiety using questionnaires and self-rating scales has been helpful in primary care settings. The Self-rating Depression Scale (SDS) and Self-rating Anxiety Scale (SAS) questionnaires have been used to evaluate depression among keratoconus patients for research purposes (15–18).

With the rapid increase in the prevalence of myopia and psychological illness, studies have begun to examine their relationship. Yokoi et al. (19) reported that about 25% of highly myopic patients had possible depression or anxiety disorders, and the presence of these psychiatric disorders was a major factor associated with low vision-related quality of life in highly myopic patients (20, 21). A study in the United Kingdom, which surveyed 112 myopic patients aged 18–65 years, reported that psychological, cosmetic, practical, and financial factors affected their quality of life (10). Li et al. (16) suggested a correlation between myopia and mental health in adolescent students, especially in terms of anxiety. Although several studies have assessed the relationship between myopia and other risk factors of depression and anxiety (16, 19, 20, 22), to the best of our knowledge, no study has tested whethervision-related risk factors are associated with the psychological well-being of university freshmen.

Therefore, this study aimed to assess the prevalence of anxiety and depression among university freshmen and to investigate the relationship between vision-related risk factors and anxiety and depression.

## Methods

### Study Design and Participants

This cross-sectional study was conducted from October 2020 to December 2020. All freshmen at the Tianjin Medical University were eligible for participation. The inclusion criteria were as follows: no eye surgery or disease, no eye trauma, and no other systematic disease. Participants who were receiving treatment from a mental health professional for either depression or anxiety disorder were excluded from the analysis. Overall, 764 of 946 students (275 men and 489 women) with a mean age of 18.2 ± 0.7 years (age range, 15–23 years) were included. This study was conducted with the approval of the authorities and the Ethics Committee of the Tianjin Medical University Eye Hospital and adhered to the tenets of the Declaration of Helsinki. Informed consent was obtained from the subjects after an explanation of the nature and possible consequences of the study. The study protocol was approved by the university.

### Eye Examination

All participants completed a detailed questionnaire concerning age, sex, height, weight, screen time, sleep time, eye disease history, and family disease history. Ophthalmic examinations of the eye were performed by an experienced ophthalmologist, namely, best corrected visual acuity (BCVA), slit-lamp examination (model YZ5X1; 66 Vision Tech Co., Ltd, Suzhou, China), non-cycloplegic autorefraction (model KR 8900; Topcon, Tokyo, Japan), lensmeter (model CL-300; Topcon, Tokyo, Japan), and ocular biometric measurements with Lenstar (LS-900; Haag-Streit AG, Köniz, Switzerland). All machine results were conducted three times to avoid bias.

Refractive errors were classified according to the spherical equivalent (SE = sphere + 0.5*cylinder) of non-cycloplegic autorefraction. Myopia was defined as an SE of −0.5 diopter (D) or less, and emmetropia was an SE between −0.50 and 0.50 D. Further classifications included mild, moderate, and high myopia as an SE of −0.5 to −3.0 D, −3.0 to −6.0 D, and <-6.0 D, respectively.

Body mass index (BMI) was calculated as weight divided by the square of height (23). We classified individuals into three BMI categories according to standard of China: lower weight (≤18.4 kg/m^2^), normal weight (18.5 kg/m^2^ ≤ BMI ≤ 23.9 kg/m^2^), and overweight (≥24.00 kg/m^2^) (24, 25).

### Questionnaires

The SAS and SDS have been widely used as simple diagnostic tools in both clinical and research settings, and their reliability and validity have been examined in the Chinese population (15–17). The higher the score on the SAS or SDS, the higher the level of mental disorder. According to the Chinese norm for the SAS and SDS, a total standard score of 53 or 50 was set as the cut-off point for depression or anxiety, respectively (15, 16).

#### Measurement of Anxiety

The SAS is a 20-item, self-reported assessment, which uses a four-point Likert scale to rate the presence and anxiety of affective symptoms and somatic components of anxiety during the previous week. Each item is scored from 1 to 4 (1, rarely; 2, occasionally; 3, frequently; and 4, always). Fifteen questions are scaled; the higher the number, the more severe the symptoms. For the remaining five questions, the lower the score, the lower the symptom severity. The level of severity of anxiety can be measured by conversion to an index score by dividing the sum of the raw score by 80 and multiplying by 100. In the Chinese public, the index score has the following two categories: no anxiety (<50) and anxiety (≥50) (16).

#### Measurement of Depression

The SDS includes 20 questions (10 positive and 10 negative). Each question is scored from 1 to 4 (1, none or a little of the time; 2, some of the time; 3, a good part of the time; and 4, most or all the time). The level of severity of depression was measured by an index equal to the SDS sum score divided by 80 and multiplying by 100. In the Chinese general population, the index had the following two categories: no depression (<53) and depression (≥53) (15).

### Statistical Analysis

All statistical analyses were performed using the SPSS statistical package for Windows (version 23.0; IBM, Armonk, NY, USA). Missing data were imputed by mean indicators. Descriptive statistics (frequencies) were calculated to assess the prevalence of anxiety and depression among the study participants. The differences between those with and without anxiety and depression were compared using Student's *t*-test for continuous variables and the chi-squared test for categorical variables. Associations between potential risk factors and the status of anxiety and depression were assessed using multivariable logistic regression, and odds ratios (ORs) and 95% CIs were calculated. General linear models were used to calculate the β coefficients and 95% CIs for the association between potential risk factors and anxiety/depression scores. Statistical significance was defined as a two-sided *P*-value of < 0.05.

## Results

### Participant Characteristics

Of 946 freshmen, 859 (response rate: 90.80%) provided informed consent to participate in the study. Of these, 95 individuals were excluded from the analysis owing to an incorrect information (*n* = 81), history of surgery (*n* = 13), and eye disease (*n* = 1). None of the students had eye trauma, systematic diseases, or were treated by doctors for mental disease. Eventually, analyses were performed on data from 764 individuals, including 489 women (64%) and 275 men (36%). The mean age for the entire sample was 18.2 ± 0.7 years (age range, 15–23 years).

The prevalence of anxiety and depression in this study was 10.34 (79/764) and 25.13% (192/764), respectively. A total of 97.9% (748/764) of participants reported a sleep time of more than 6 h/day and 70.54% (539/764) had more than 4 h/day of screen time. A total of 59.42% (454/764) of students had a BMI within the normal range according to Chinese standards. A total of 71.60% (547/764) of students had an axial length (AL) longer than 26 mm, which was considered the threshold for higher risk of myopia. A total of 26.70% (204/764) of students had myopia higher than 6.00 D. However, only 19.50% (149/764) of students wore glasses more than 6.00 D; that is, the glasses they wore were undercorrected. The mean BCVA of 0.87 ± 0.26 reflected the same result.

Sleep time was different between participants with and without anxiety and (*P* < 0.05). In those with >7 h/day of sleep, the percentage of students with anxiety was much lower than that of those without anxiety (59.49 vs. 68.47%), while the percentage of students with anxiety was higher than those who did not suffer from anxiety in those with <6 h/day of sleep (2.54 vs. 2.04%). There were no significant differences in the distributions of age, sex, screen time, BMI, AL, SE, spectacle power, or BCVA between the two groups. The same results were found in the depression group (Table 1).

**Table 1:** Characteristics of the participants with and without anxiety, depression in Tianjin Medical University in 2020.

		**Anxiety**			**Depression**		
		**No, 685**	**Yes, 79**		**No, 572**	**Yes, 192**	
		***N***	***N***	***p***	***N***	***N***	***p***
Age	<18 y	521 (76)	62 (78.48)	0.632	435 (76.05)	148 (77.08)	0.771
	≥18 y	164 (23.94)	17 (21.52)		137 (23.95)	44 (22.92)	
Gender	Female	431 (62.9)	58 (73.42)	0.066	368 (64.33)	121 (63.02)	0.743
	Male	254 (37.08)	21 (26.58)		204 (35.66)	71 (36.98)	
BMI (kg/m^2^)	≤ 18.4	141 (20.58)	21 (26.58)	0.272	120 (20.98)	42 (21.88)	0.751
	18.5–23.9	406 (59.27)	48 (60.76)		339 (59.27)	115 (59.90)	
	≥24	128 (18.69)	10 (12.66)		107 (18.71)	31 (16.15)	
Sleep time (h/d)	>7	469 (68.47)	47 (59.49)	0.05^*^	373 (65.21)	143 (74.48)	0.07
	6–7	202 (29.49)	30 (37.97)		187 (32.69)	45 (23.44)	
	<6	14 (2.04)	2 (2.54)		12 (2.10)	4 (2.18)	
Screen time (h/d)	2–4	205 (29.93)	20 (25.32)	0.071	168 (29.37)	57 (29.69)	0.837
	4–6	398 (58.10)	55 (69.62)		342 (59.79)	111 (57.81)	
	6–8	59 (8.61)	1 (1.27)		42 (7.34)	18 (9.38)	
	>8	7 (1.02)	1 (1.27)		6 (1.05)	2 (1.04)	
BCVA		0.86 (0.26)	0.90 (0.23)	0.303	0.88 (0.26)	0.84 (0.25)	0.119
AL (mm)	<26	493 (71.97)	54 (68.35)	0.462	408 (71.33)	139 (72.40)	0.854
	>26	189 (27.59)	25 (31.65)		161 (28.15)	53 (27.60)	
SE (D)	>+0.50	11 (1.61)	1 (1.27)	0.233	9 (1.57)	3 (1.56)	0.268
	−0.50 to +0.50	48 (7.01)	1 (1.27)		35 (6.12)	14 (7.29)	
	−0.50 to −3.00	164 (23.94)	16 (20.25)		145 (25.35)	35 (18.23)	
	−3.00 to −6.00	281 (41.02)	38 (48.10)		238 (41.61)	81 (42.19)	
	< -6.00	181 (26.42)	23 (29.11)		145 (25.35)	59 (30.73)	
Spectacle power (D)	>-3.00	283 (41.31)	25 (31.65)	0.219	236 (41.26)	72 (37.5)	0.721
	−3.00 to −6.00	260 (37.96)	34 (43.04)		218 (38.11)	76 (39.58)	
	<-6.00	130 (18.98)	19 (24.05)		110 (19.23)	39 (20.31)	

* *p < 0.05*.

*N, number; BMI, Body Mass Index; BCVA, Best Corrected Visual Acuity; AL, axial length; SE, Spherical Equivalent; D, diopters*.

### Associations of Demographic, Lifestyle, and Vision Characteristics With Anxiety and Depression

All risk factors were entered into multivariable logistic regression models. Table 2 shows the results of multivariable logistic regression analyses of the risk factors associated with the prevalence of anxiety and depression symptoms. We found that spectacle power (OR = 0.89; 95% CI: 0.81–0.98; *P* = 0.019), SE (OR = 0.89; 95% CI: 0.81–0.98; *P* = 0.025), sleep time (OR = 0.53; 95% CI: 0.35–0.79; *P* = 0.002), and BMI (OR = 0.93; 95% CI: 0.86–0.99; *P* = 0.047) were significantly associated with anxiety status. No significant association was observed between any of the variables and depression. SE [variance inflation factor (VIF) = 3.874], AL (VIF = 1.694), and spectacle power were not included in the model together to avoid collinearity.

**Table 2:** Associations of demographic, lifestyle and vision characteristics with anxiety, and depression status (*n* = 764).

	**Anxiety**		**Depression**	
	**OR (95%CI)**	***p***	**OR (95%CI)**	***p***
Spectacle power (D)	**0.89 (0.81, 0.98)**	0.019^*^	1.00 (0.93, 1.07)	0.942
SE (D)^†^	**0.89 (0.81, 0.98)**	0.025^*^	0.98 (0.91, 1.04)	0.473
AL (mm)^‡^	1.20 (0.98, 1.48)	0.08	1.03 (0.89, 1.19)	0.675
Age, yrs	0.84 (0.56, 1.26)	0.399	1.05 (0.81, 1.36)	0.704
Gender	0.60 (0.34, 1.06)	0.076	1.06 (0.74, 1.54)	0.744
BMI (kg/m^2^)	**0.93 (0.86, 0.99)**	0.047^*^	0.99 (0.94, 1.03)	0.542
Sleep time (h/d)	**0.53 (0.35, 0.79)**	0.002^*^	1.26 (0.95, 1.69)	0.112
Screen time (h/d)	0.91 (0.72, 1.14)	0.411	1.03 (0.89, 1.20)	0.653
BCVA	2.25 (0.84, 6.03)	0.108	0.58 (0.30, 1.13)	0.108

* *p < 0.05*.

*OR, odds ratio; CI, confidence interval; SE, Spherical Equivalent; D, diopters; AL, axial length; BMI, Body Mass Index; BCVA, Best Corrected Visual Acuity*.

*Model 1 adjusted for age, gender, BMI, sleep time, screen time, BCVA, and glass diopters*.

† *Model 2 adjusted for age, gender, BMI, sleep time, screen time, BCVA, and SE*.

‡ *Model 3 adjusted for age, gender, BMI, sleep time, screen time, BCVA, and AL*.

In Model 1, spectacle power was negatively correlated with anxiety scores (β = −0.13, *P* = 0.001). In Model 2, the anxiety score decreased by 0.11 per unit increase of SE (*P* < 0.006). In Model 3, AL was positively associated with anxiety scores (β = 0.08, *P* < 0.047). Other risk factors were not associated with anxiety scores (*P* > 0.05). As for depression, there was only a significant association between sleep time and depression scores (β = 0.12, *P* = 0.002), while other risk factors were not related to depression scores (Table 3).

**Table 3:** Associations of demographic, lifestyle and vision characteristics with anxiety, and depression score (*n* = 764).

	**Anxiety**		**Depression**	
	**β (95%CI)**	***p***	**β (95%CI)**	***p***
Spectacle power (D)	**−0.13 (−0.67**, **−0.18)**	0.001*	0.19 (−0.11, 0.18)	0.619
SE (D)^†^	–**0.11 (**–**0.60**, –**0.10)**	0.006*	−0.04 (−0.23, 0.06)	0.259
AL (mm)^‡^	**0.08 (0.01, 1.07)**	0.047*	−0.01 (−0.35, 0.27)	0.786
Age, yrs	−0.01 (−1.11, 0.77)	0.725	−0.01 (−0.59, 0.51)	0.884
Gender	−0.04 (−2.07, 0.58)	0.272	0.00 (−0.77, 0.79)	0.986
BMI (kg/m^2^)	−0.05 (−0.30, 0.05)	0.153	−0.03 (−0.14, 0.06)	0.460
Sleep time (h/d)	−0.07 (−1.99, 0.07)	0.068	**0.12 (0.37, 1.58)**	0.002*
Screen time (h/d)	0.01 (−0.44, 0.62)	0.734	−0.00 (−0.33, 0.29)	0.908
BCVA	0.02 (−1.71, 3.09)	0.573	−0.04 (−2.12, 0.69)	0.319

* *p < 0.05*.

*SE, Spherical Equivalent; D, diopters; AL, axial length; BMI, Body Mass Index; BCVA, Best Corrected Visual Acuity*.

*Model 1 adjusted for age, gender, BMI, sleep time, screen time, BCVA, and glass diopters*.

† *Model 2 adjusted for age, gender, BMI, sleep time, screen time, BCVA, and SE*.

‡ *Model 3 adjusted for age, gender, BMI, sleep time, screen time, BCVA, and AL*.

## Discussion

The results of the current study indicate that the overall prevalence of anxiety and depression was 10.34 and 25.13%, respectively, among Chinese freshmen during the COVID-19 pandemic. There were significant associations between anxiety status and spectacle power, SE, sleep time, and BMI in logistic regression. In three multivariable linear regression models, spectacle power and SE were negatively associated with anxiety scores, whereas AL was positively related to anxiety scores. For every 1 h decrease in sleep time, the depression score is increased by 0.12. Findings from this study indicate that myopia was associated with both anxiety status and anxiety score, regardless of the index used. SE, AL, and spectacle power are all markers that reflect the severity of myopia, where SE is the real refractive error, AL is the length of the eyeball, and spectacle power is the degree of correction with glasses. The lower the SE or spectacle power, the higher the myopia. The longer the AL, the higher the myopia (2, 4, 16, 26). In the current study, the likelihood of anxiety increased per unit of spectacle power, and SE decreased. Spectacle power and SE were negatively associated with anxiety scores, while AL was positively associated with the score.

Previous studies have focused on the relationship between high myopia and mental health (19, 20); however, we assessed emmetropia, mild myopia, moderate myopia, and high myopia in our study. Most studies investigated the relationship in adolescents (12, 19–22, 27), and few studies are available on university students. Previous studies have shown that adolescents with myopia are more likely to suffer from psychological problems than their peers (12, 16, 20), while we assessed whether this situation still exists when they mature. Moreover, most studies have focused on social risk factors, such as sex and family income, and few have investigated the vision-related risk factors of anxiety or depression (10, 19, 27). We investigated the prevalence and vision-related risk factors of anxiety and depression among university freshmen.

In a high school student population, Li et al. (16) found that spectacle power was associated more closely with anxiety than depression in 1st-year high school students. When the spectacle power increased by 0.0848 D, the SAS scores increased by 1 point. Our results are consistent with those of Li et al.

Why do myopic students seem more anxious? Seitler (28) suggested that myopia is a refractive error caused by muscle tension outside the eye, causing a break in the separation-individuation process in which myopic patients experience separation anxiety resulting in a sense of an inability to cope with the world.

Furthermore, myopic students who wore glasses were bullied at school and felt victimized. Suffering in victims of bullying occurs due to stressful situations and dismissal to the margin of the group and a low social status among their peers (29). Copeland et al. (30) found that victims of bullying are at risk for psychiatric problems, and this risk extends into early adulthood. Meanwhile, myopia, especially high myopia, can decrease the quality of life (10). The quality of life of patients with high myopia is significantly lower than that of patients without myopia (10). A decreased quality of life affects the psychological status of patients.

In our study, most university students wore glasses to correct their myopia, which may also partly explain their anxiety. Prior research (31) has indicated that the myopic children wearing contact lenses evaluate their physical appearance, athletic skills, and social interactions more favorably than those with glasses, as glasses reduce the size of the eye and affect appearance. High myopia, defined as AL ≥ 26 mm, may drastically increase the risk of severe complications later in life, namely, myopic maculopathy, retinal detachment, and glaucoma, which can cause blindness (32). As a result, individuals with high myopia and a longer AL live in fear of possible blindness in the future, which may induce anxiety.

In the current study, we found that the prevalence of anxiety and depression was 10.34 and 25.13%, respectively. The rates were slightly lower than that of Mao (13), who reported that the mean prevalence of anxiety was 27.22% among medical students in China, while the mean prevalence of depression was 32.74%. The discrepancy across studies may be due to the following reasons. First, different assessment tools and criteria have been used in different articles. For example, Mao used the Beck Anxiety Inventory and Hamilton Anxiety Scale (33) to define anxiety and the Beck Depression Inventory (34) and the Center for Epidemiologic Studies Depression Scale (33) to define depression. Second, our participants were university freshmen aged 18–21 years, while Mao focused on both undergraduate and graduate students. The 5th-year undergraduate students and 3rd-year graduate students were facing employment pressure and feared future uncertainty.

Complaints of poor sleep were reported in up to 90% of people diagnosed with depression (35) and up to 70% of people with anxiety (36). Our results are in line with those of previous reports. We found that students with anxiety slept less than their peers did. Sleep time was also associated with depression score: the lower the sleep time, the higher the depression score.

Screen time was not significantly correlated with anxiety or depression. In contrast, Maras et al. (37) examined 2,482 grade 7–12 students and concluded that screen time was associated with the severity of depression and anxiety. It is plausible that the age difference could explain this inconsistency. Adolescents have limited self-control and feel frustrated once they decrease screen time, whereas university freshmen are adults with presumably more self-control. The percentage of students who had >6 h/day of screen time was only 8.9% during the COVID-19 pandemic.

Nevertheless, the percentage of students with screen time of more than 6 h/day was higher than that in a previous study conducted before COVID-19 (38). Therefore, we inferred that the increased screen time was caused by the COVID-19 pandemic. The increased near work at home and limited outdoor activities were all found to be associated with the progression of myopia, and myopia severity could be aggravated during and beyond the COVID-19 pandemic period. The pandemic may last for a relatively short time, but the negative impact of myopia on mental health may last for a long time. Schools, parents, doctors, and students themselves should create a joint response to these challenges.

This study has some limitations. First, because of the cross-sectional design of this study, causality cannot be clarified. Second, sleep time and screen time were self-reported; therefore, recall and reporting bias cannot be excluded. Third, the subjects recruited from a large Chinese university were generally healthy and well-educated, and one should be cautious in generalizing our findings to Chinese young adults. Finally, although we have adjusted some predictors such as screen time and sleep time, other factors (such as the changes that occur during the transition from school life to university life, changes in campus life during the lockdown, and technical stress resulting from online education) not surveyed in this study may also confound the association between myopia and anxiety and depression symptoms.

## Conclusions

Myopia was associated with anxiety and anxiety scores. The higher the myopia, the higher the anxiety score. Myopia was not found to be associated with depression. The results highlight the importance of providing psychological support to students with myopia during the COVID-19 pandemic.

## Data Availability Statement

The raw data supporting the conclusions of this article will be made available by the authors, without undue reservation.

## Ethics Statement

The studies involving human participants were reviewed and approved by Tianjin Medical University Eye Hospital. The patients/participants provided their written informed consent to participate in this study.

## Author Contributions

RW and HY conceived and supervised the experiment. HZ, HG, WD, and YiZ performed the study and collected the data. YuZ analyzed the data. HZ and YuZ wrote the manuscript. All authors read and approved the final manuscript.

## Funding

This work was supported by a grant from the Science and Technology Development Fund of Tianjin Education Commission for Higher Education of China (No. 2018KJ056).

## Conflict of Interest

The authors declare that the research was conducted in the absence of any commercial or financial relationships that could be construed as a potential conflict of interest.

## Publisher's Note

All claims expressed in this article are solely those of the authors and do not necessarily represent those of their affiliated organizations, or those of the publisher, the editors and the reviewers. Any product that may be evaluated in this article, or claim that may be made by its manufacturer, is not guaranteed or endorsed by the publisher.
